# Enhanced Electroactive Phases of Poly(vinylidene Fluoride) Fibers for Tissue Engineering Applications

**DOI:** 10.3390/ijms25094980

**Published:** 2024-05-02

**Authors:** Angelika Zaszczyńska, Arkadiusz Gradys, Anna Ziemiecka, Piotr K. Szewczyk, Ryszard Tymkiewicz, Małgorzata Lewandowska-Szumieł, Urszula Stachewicz, Paweł Ł. Sajkiewicz

**Affiliations:** 1 Laboratory of Polymers Biomaterials, Institute of Fundamental Technological Research, Polish Academy of Sciences, Pawińskiego 5B, 02-106 Warsaw, Poland; azasz@ippt.pan.pl (A.Z.); argrad@ippt.pan.pl (A.G.); rtym@ippt.pan.pl (R.T.); 2 Laboratory of Cell Research and Application, 02-106 Warsaw, Poland; ania.cieciuch@gmail.com (A.Z.); malgorzata.lewandowska-szumiel@wum.edu.pl (M.L.-S.); 3 Faculty of Metals Engineering and Industrial Computer Science, AGH University of Krakow, 30-059 Krakow, Poland; pszew@agh.edu.pl (P.K.S.); ustachew@agh.edu.pl (U.S.); 4 Department of Histology and Embryology, Centre for Biostructure Research, Medical University of Warsaw, Centre for Preclinical Research and Technology, 02-106 Warsaw, Poland

**Keywords:** scaffolds, polymers, piezoelectricity, bone tissue engineering, nanofibers, regenerative medicine

## Abstract

Nanofibrous materials generated through electrospinning have gained significant attention in tissue regeneration, particularly in the domain of bone reconstruction. There is high interest in designing a material resembling bone tissue, and many scientists are trying to create materials applicable to bone tissue engineering with piezoelectricity similar to bone. One of the prospective candidates is highly piezoelectric poly(vinylidene fluoride) (PVDF), which was used for fibrous scaffold formation by electrospinning. In this study, we focused on the effect of PVDF molecular weight (180,000 g/mol and 530,000 g/mol) and process parameters, such as the rotational speed of the collector, applied voltage, and solution flow rate on the properties of the final scaffold. Fourier Transform Infrared Spectroscopy allows for determining the effect of molecular weight and processing parameters on the content of the electroactive phases. It can be concluded that the higher molecular weight of the PVDF and higher collector rotational speed increase nanofibers’ diameter, electroactive phase content, and piezoelectric coefficient. Various electrospinning parameters showed changes in electroactive phase content with the maximum at the applied voltage of 22 kV and flow rate of 0.8 mL/h. Moreover, the cytocompatibility of the scaffolds was confirmed in the culture of human adipose-derived stromal cells with known potential for osteogenic differentiation. Based on the results obtained, it can be concluded that PVDF scaffolds may be taken into account as a tool in bone tissue engineering and are worth further investigation.

## 1. Introduction

We are witnessing a rapid aging of society. More and more people are hospitalized due to bone system diseases [1]. Despite the great achievements of modern medicine, many diseases are still not effectively treated. Approximately 1.5 million individuals suffer from bone diseases such as osteoporosis [2]. Furthermore, bone fractures represent a prevalent musculoskeletal issue necessitating hospitalization more frequently than any other condition in this category [3]. The challenges are colossal, and several solutions must be implemented to improve patient care. Intensive work on materials for medicine that will support the regeneration of bone tissue is necessary.

**Figure 1:**
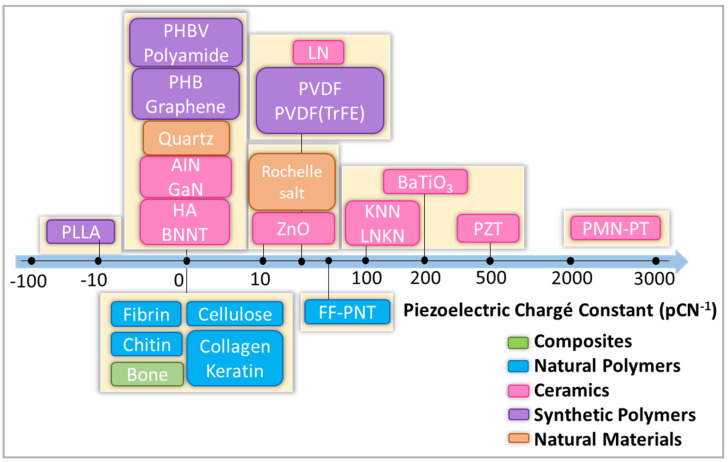
Classification of piezoelectric materials. P(VDF-TrFE): poly(vinylidene fluoride-trifluoro ethylene), PVDF: poly(vinylidene fluoride), PHB: polyhydroxybutyrate, PLLA: poly(l-lactic acid), gallium nitride (GaN), boron nitride (BN), zinc oxide (ZnO), HA: hydroxyapatite.

Bone tissue regeneration is a complex biological phenomenon. Many material requirements must be fulfilled. In the realm of biomedical engineering, there has been a growing surge of scientific and technical attention towards investigating smart materials (SM) over the past few decades. These materials have garnered interest due to their ability to react to different external stimuli, including physical, chemical, and mechanical signals, thereby mimicking the behavior observed in natural bodily tissues [4]. One important type of SM is piezoelectric scaffolds, which can generate electrical signals in response to the applied stress. Furthermore, they can stimulate the signaling pathways and thereby enhance tissue regeneration at the impaired site [5]. Piezoelectric phenomena are observed in various molecular structures in animal bodies, for example, in DNA, collagen, and keratin, being present in multiple tissues and organs like dentin, tendons, and, most importantly, in human bones [6]. The existence of piezoelectricity in the living organism was shown first by Fukada et al. [7] in 1957 (dry and wet bones) and Basset in 1962 [8]. He described the generation of electrical potentials by bone responses to mechanical stress. It is known that electrical charges are important for the activity of cells, particularly for human adipose-derived stromal cells [9]. One significant benefit of piezoelectric scaffolds is their capacity to generate electrical potential in a non-invasive manner when subjected to mechanical forces, eliminating the requirement for invasive electrodes. In bone tissue engineering, the main properties of the scaffolds are accounted for to emulate both the morphology and function of the native tissue. Consequently, piezoelectric scaffolds can dynamically mimic the cellular microenvironment to be appropriate for a specific bone tissue engineering injury [10,11,12]. The presence of polar phases combined with fiber morphology and relatively high elasticity provides the perspective of using this material in bone regeneration [13]. Classification of various piezoelectric materials is shown in Figure 1. 

**Figure 2:**
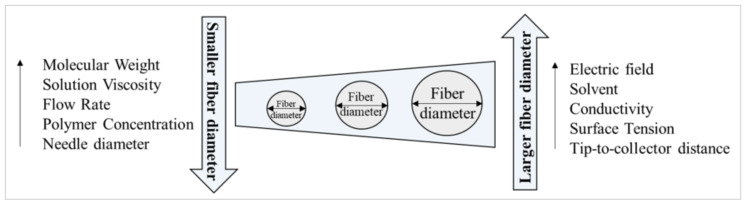
Schematic representation of the influence of electrospinning parameters on fiber diameter.

The important property of nanofibrous piezoelectric scaffolds is biocompatibility, but also topography, porosity, density, geometry [14,15], humidity [16], and electrical polarity [17]. In the last few years, several studies have shown that different techniques, such as phase separation, 3D printing, photolithography, and electrospinning, have been tested to create a unique 3D nanofibrous scaffold with appropriate features [18]. The general influence of electrospinning parameters on fiber diameter is shown in Figure 2.

**Figure 3:**
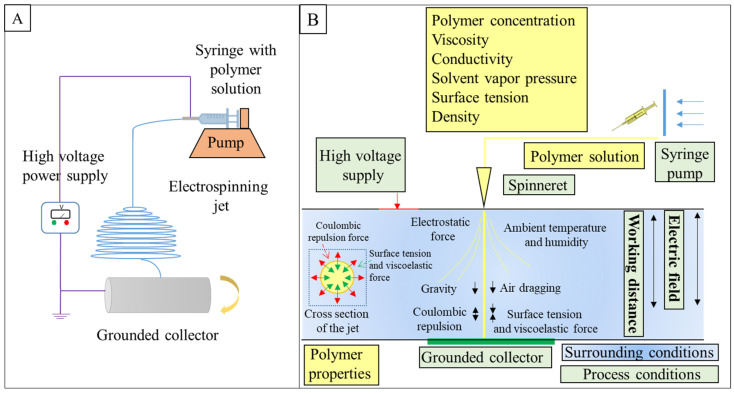
(**A**) Schematic illustration of electrospinning setup, (**B**) general scheme of nanofiber formation using electrospinning.

In tissue engineering, electrospinning is widely investigated as a scaffold for drug delivery [19], bone tissue engineering [20], cardiac tissue engineering [21], cartilage [22], soft tissue [23], or skin [24]. In addition to many advantages of electrospinning, such as the controlled architecture of the scaffolds providing similarity to ECM, cheapness, and simplicity [25], the electrospinning technique (Figure 3A) is a particularly promising method of PVDF scaffold formation because of the possibility of reaching relatively high polar form and thus piezoelectricity by optimizing electrospinning conditions [26]. The electrospinning configuration comprises a high-voltage power source, a spinneret assembly, and a grounded collector. The spinneret part contains a syringe with a needle, feed storage that is polymer solution, and a special pump, which injects solution at a constant flow rate. High voltage is connected to the tip of the metal needle, thereby generating electrostatic forces [27], which in addition to surface tension, viscoelastic force, air drag, and gravity force, result in the formation of the nanofibers from a liquid jet (Figure 3B). The main stages of the electrospinning process are the starting formation of the cone at the top of the spinneret and the development of a rectilinear jet; evaporation of solvent during the bending deformation with looped and spiral trajectories during the process; final nanofiber collections on the collector [28,29].

**Figure 4:**
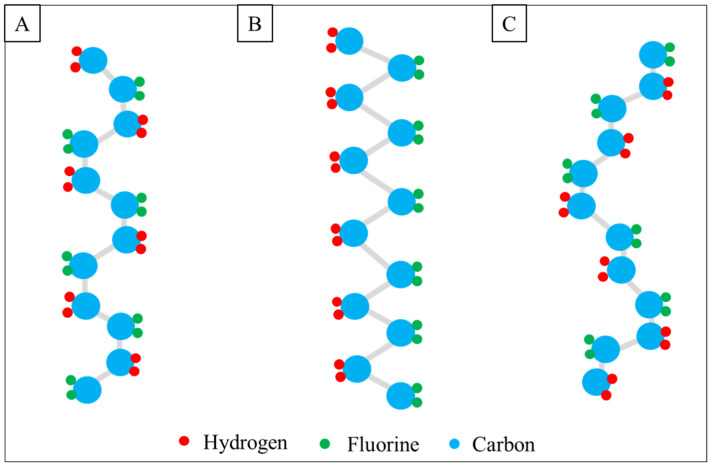
Schematic representation of the chain conformation of the phases in PVDF; (**A**)—α-phase, (**B**)—β-phase, and (**C**)—γ-phase.

Various natural and synthetic polymers have been investigated for bone tissue engineering applications [30]. One of the most promising is piezoelectric PVDF with strong chemical resistance, biocompatibility, and optical transparency [31]. PVDF is a semi-crystalline and can crystallize in at least five different crystallographic phases: α, β, γ, δ, and ε (Figure 4). They differ in chain conformation and arrangement of CH_2_–CF_2_ dipoles, resulting in various net dipole moments. A strong electric moment in the PVDF monomer unit arises because of the strong electro-negativity of fluorine atoms as compared to hydrogen atoms. In case the polymer chains are packed into crystals to form parallel dipoles, the crystal has a non-zero net dipole moment. Observations indicate distinct characteristics across the β, γ, and δ phases. The β phase exhibits the highest dipole moment, primarily attributed to its all-trans conformation. Conversely, parallel dipole arrangements are evident in alternative chain conformations such as TGTG^−^ and T3GT3G^−^, corresponding to lower polarity in the δ and γ phases, respectively. Conversely, identical conformations result in a net zero dipole moment due to antiparallel chain arrangements, as seen in the α and ϵ phases [32]. Thus, the most electrically important polymorphic variant due to the highest piezo-, ferro- and pyroelectric properties is the β-phase [33]. In the recent literature, the electrospinning technique has been quite frequently used for the formation of PVDF fibers. It is known that the electrospinning of PVDF from solutions generally favors the formation of polar phases [34]. It is explained by the extremely short time of solvent evaporation being equivalent to fast cooling in melt crystallization as well as the electric interactions of the polymer with the external electric field [35]. In the case of pure PVDF, Cozza et al. [36] have revealed that the content of polar β-phase is much higher in electrospun fibers than cast film. As such, systematic studies from the perspectives of polymer molecular weight and electrospinning parameters are necessary.

The formation of random and aligned fiber orientation scaffolds allows the forming of connected 3D nonwovens of micro- and nano-fibers with high surface area and porosity, which should enhance cell adhesion [37]. Hence, fibrous scaffolds may offer a more conducive environment for cellular attachment and proliferation compared to non-fibrous scaffolds [38]. In this study, PVDF cell scaffolds in the form of nanofibers (lowly and highly oriented) were formed by the electrospinning technique. The effect of PVDF molecular masses, fiber spatial arrangements of fibers, and electrospinning parameters during the process on polymorph content was investigated. The aim of the research was to optimize the morphology, chemical structure, content of electroactive phases, piezoelectricity coefficient, and biocompatibility from the perspective of material suitable for BTE.

## 2. Results and Discussion

### 2.1. Morphology

**Figure 5:**
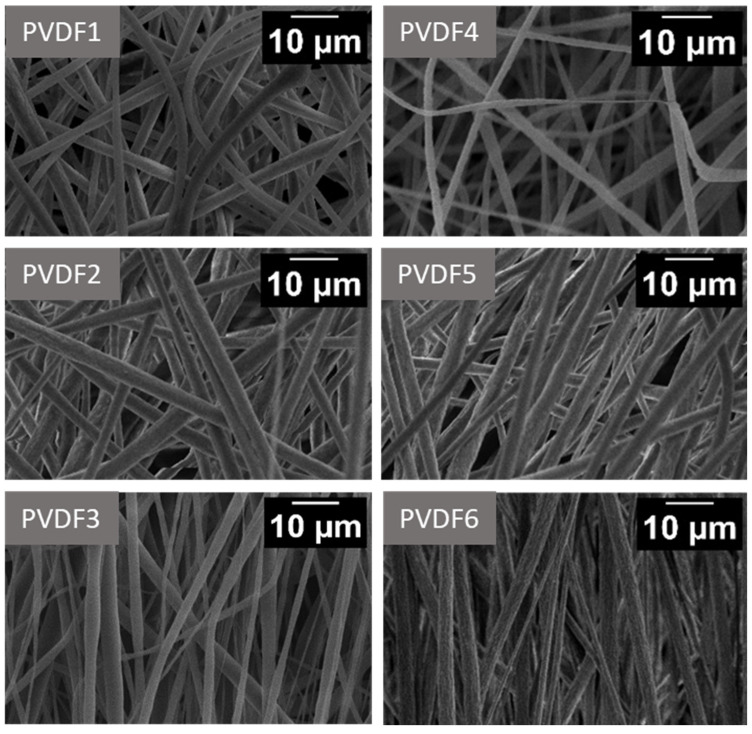
SEM images of electrospun fiber mats. PVDF1—180,000 g/mol, collector speed—200 rpm; PVDF2—180,000 g/mol, collector speed—1000 rpm; PVDF3—180,000 g/mol, collector speed—2000 rpm; PVDF4—530,000 gm/mol, collector speed—200 rpm; PVDF5—530,000 g/mol, collector speed—1000 rpm; PVDF6—530,000 g/mol, collector speed—2000 rpm. All samples C_p_ = 20%, F_R_—1 mL/h (details in Table 4).

**Figure 6:**
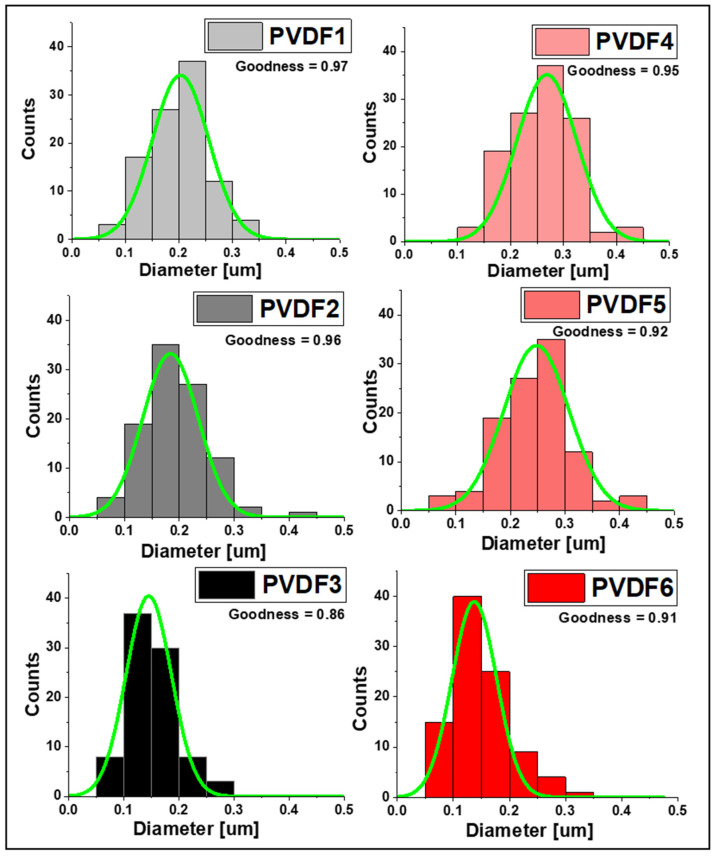
Histograms of the PVDF fiber diameter distribution approximated with the Gauss function. PVDF1—180,000 g/mol, collector speed—200 rpm; PVDF2—180,000 g/mol, collector speed—1000 rpm; PVDF3—180,000 g/mol, collector speed—2000 rpm; PVDF4—530,000 gm/mol, collector speed—200 rpm; PVDF5—530,000 g/mol, collector speed—1000 rpm; PVDF6—530,000 g/mol, collector speed—1000 rpm. All samples Cp = 20%, F_R_—1 mL/h (details in Table 4).

**Figure 7:**
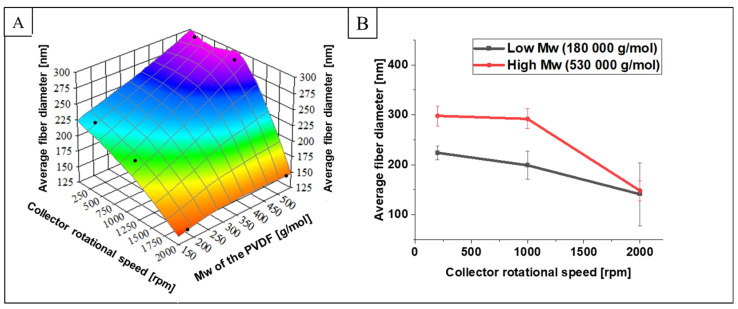
The relation between collector rotational speed, polymer molecular weight, and average fiber diameter (**A**), and relation between collector rotational speed and average fiber diameter (**B**). PVDF1—180,000 g/mol, collector speed—200 rpm; PVDF2—180,000 g/mol, collector speed—1000 rpm; PVDF3—180,000 g/mol, collector speed—2000 rpm; PVDF4—530,000 gm/mol, collector speed—200 rpm; PVDF5—530,000 g/mol, collector speed—1000 rpm; PVDF6—530,000 g/mol, collector speed—1000 rpm. All samples Cp = 20%, F_R—_1 mL/h (details in Table 4).

SEM images of fiber mats collected at 200, 1000, and 2000 rpm of the collector speed for the two Mws (Figure 5) indicate continuous bead-free morphology of the nanofibers. It is evident from Figure 6 that the average fiber diameter decreases with increasing collector rotational speed. The reduction in diameter is particularly evident when changing rotational speed from 1000 to 2000 rpm. From Figure 7 it can be concluded that at the high rotational speed of the collector, the average diameter does not depend on the molecular weight. In contrast, at low rotational speeds of the collector, the influence of the molecular weight on the average diameter of the fibers is observed. In conditions of low rotational speed, when the fiber stretching force exerted by the collector on which the fiber is wound is low, the effect of molecular weight on the fiber diameter is visible. It is caused by the density of chain splicing, which is higher for large molecular weights, at which the viscosity is high, and the viscous resistance during fiber formation is high. As the collector rotates rapidly, the tensile (mechanical) forces eliminate any viscosity issues, and thin fibers are formed due to the significant mechanical field from the collector [39,40]. In the case of low molecular weight PVDF, the total porosity was 82 ± 0.16%, 80 ± 0.22%, and 79 ± 0.4% for PVDF1, PVDF2, and PVDF3, respectively, and the mean pore size was ~215 ± 12 nm. The porosity increased with the increase in molecular weight [41] and was 84 ± 0.11%, 83 ± 0.13%, and 81 ± 0.45% for samples PVDF4, PVDF5, and PVDF6, respectively, and the mean pore size was ~189 ± 19 nm.

Also, the average diameter is higher for PVDF6 is 148 ± 84 nm (Mw—530,000 g/mol), compared to samples with lower molecular weight (Mw—180,000 g/mol) and the same collector speed (PVDF3 = 141 ± 62 nm). More precisely, the slight diameter reduction is connected to tangential force, which increases with molecular weight and the higher rotating speed of the collector. Generally, in random and aligned samples, the diameter of the fibers decreases with higher collector rotational speed, combined the effect of collector rotational speed and polymer molecular weight is interdependent. Additionally, the dimensions of the fibers are lower in samples with higher collector rotational speed in random and aligned orientations, which is in agreement with the literature [42]. Higher rotational speed can partially counteract the hindered elongation caused by higher molecular weight, leading to a reduction in average fiber diameter. However, the dominant factor affecting fiber diameter is often the molecular weight of the polymer.

**Figure 8:**
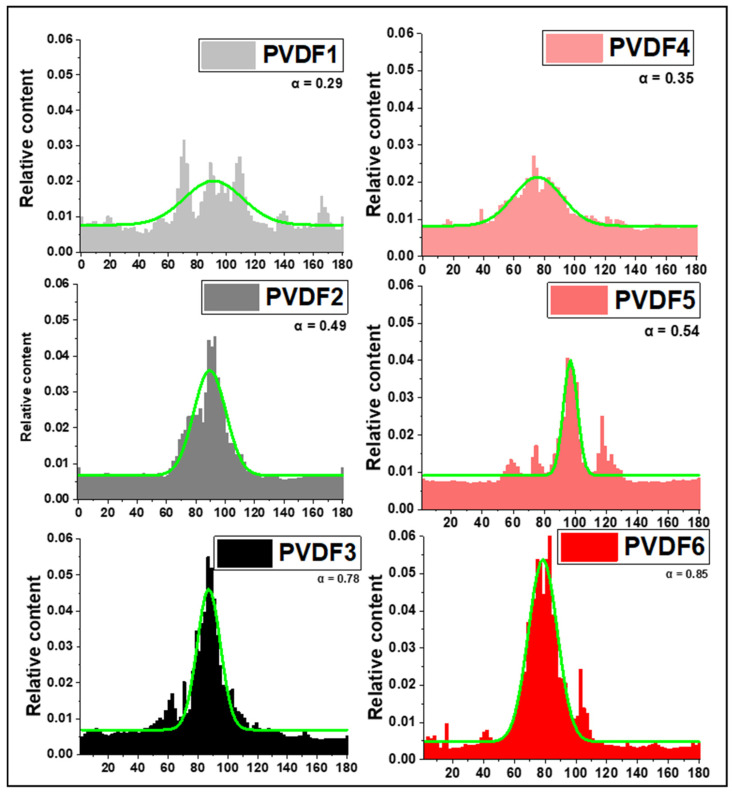
The distribution of PVDF nanofibers orientation, the anisotropy index (α) calculated from Equations (1) and (2). PVDF1—180,000 g/mol, collector speed—200 rpm; PVDF2—180,000 g/mol, collector speed—1000 rpm; PVDF3—180,000 g/mol, collector speed—2000 rpm; PVDF4—530,000 gm/mol, collector speed—200 rpm; PVDF5—530,000 g/mol, collector speed—1000 rpm; PVDF6—530,000 g/mol, collector speed—1000 rpm. All samples Cp = 20%, F_R_—1 mL/h (details in Table 4).

**Figure 9:**
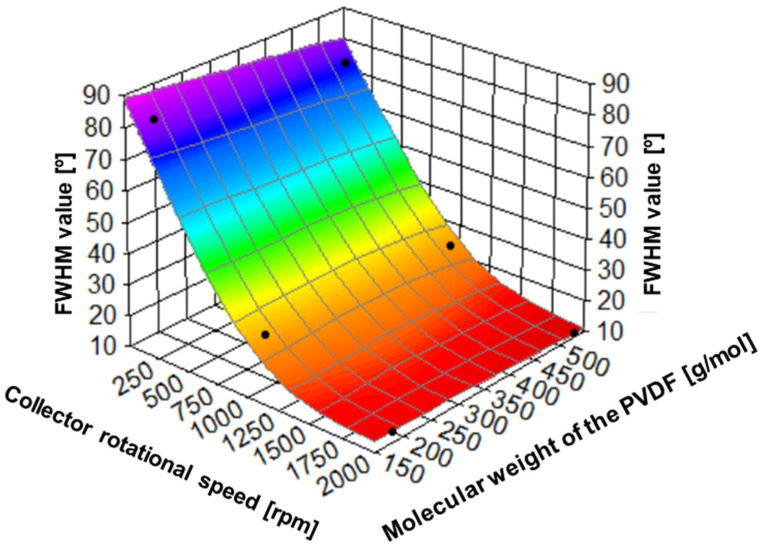
The relation between collector rotational speed, polymer molecular weight, and fiber alignment (expressed as FWHM values).

The effect of collector rotational speed and polymer molecular weight on spatial arrangements of fibers can be deduced from the analysis of the orientation distribution of PVDF (Figure 8), and FWHM (Figure 9). It is evident that the fiber orientation becomes higher with the increasing speed of the rotational collector, being weakly dependent on the molecular weight. The full width at half maximum (FWHM) of the orientation of the fibers changes from 81.30 o at 200 rpm, through 27.13 at 1000 rpm, and 13.29 at 2000 rpm in PVDF for lower molecular weight PVDF, and 74.10 o at 200 rpm, through 25.19 at 1000 rpm, and 11.16 at 2000 rpm for higher molecular weight PVDF. Higher rotational speeds lead to increased tensile forces, which can enhance the stretching and alignment of the fibers [43]. To quantitatively compare the effect of the rotational speed of the collector on the arrangement of PVDF nanofibers, the anisotropy index was used (α). As the rotating speed increases from 200 to 2000 rpm, the α increases from 0.29 to 0.78 in PVDF with low molecular weight and from 0.35 to 0.84 in PVDF with higher molecular weight. It indicates clearly an increase in the nanofibers’ orientation with the rotational speed of the collector. The comparison of the anisotropy index for different molecular weights shows only a small increase in orientation with increasing molecular weight.

### 2.2. Influence of the Process Parameters on the Electroactive Phases Content

#### 2.2.1. Collector Rotational Speed

**Figure 10:**
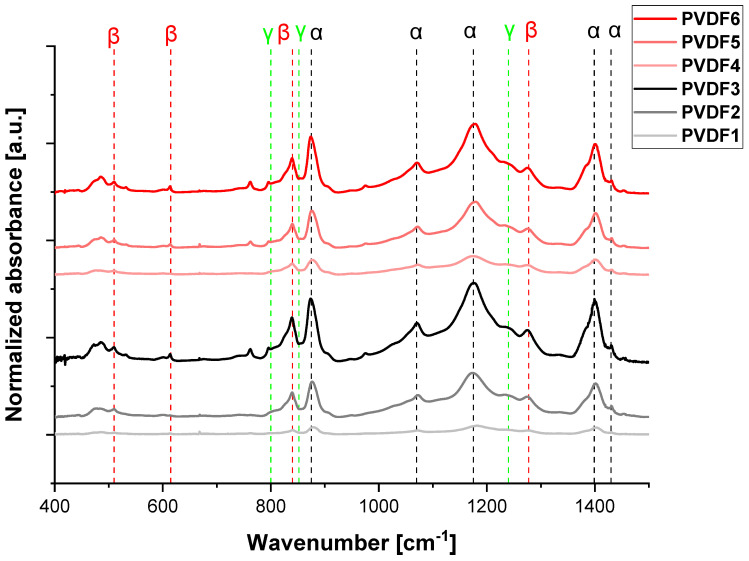
FTIR-ATR spectrum (400–1500 cm^−1^) of selected samples with various molecular weights and rotational speeds of the collector. PVDF1—180,000 g/mol, collector speed—200 rpm; PVDF2—180,000 g/mol, collector speed—1000 rpm; PVDF3—180,000 g/mol, collector speed—2000 rpm; PVDF4—530,000 gm/mol, collector speed—200 rpm; PVDF5—530,000 g/mol, collector speed—1000 rpm; PVDF6—530,000 g/mol, collector speed—1000 rpm. All samples Cp = 20%, F_R_—1 mL/h (details in Table 4).

Figure 10 shows the FTIR spectra of the nanofibrous samples. The bands at 490, 766, 1402, and 1432 cm^−1^ are assigned to the α phase, while the bands at 510, 600, 840, and 1280 cm^−1^ are assigned to the electroactive β-phase. Additionally, the bands at 812, 840, and 1234 cm^−1^ correspond to the electroactive γ-phase. Some of the α and β bands are overlapped with the bands coming from the γ-phase, for instance, with the band at 840 cm^−1^. Multiple phases such as α, β, and γ phases coexist with partially overlapped bands. The relative amount of β-phase in each sample was estimated using the absorption bands at 840 and 766 cm^−1^ (corresponding to the β and α phases) and calculated from Equations (3)–(5).

**Figure 11:**
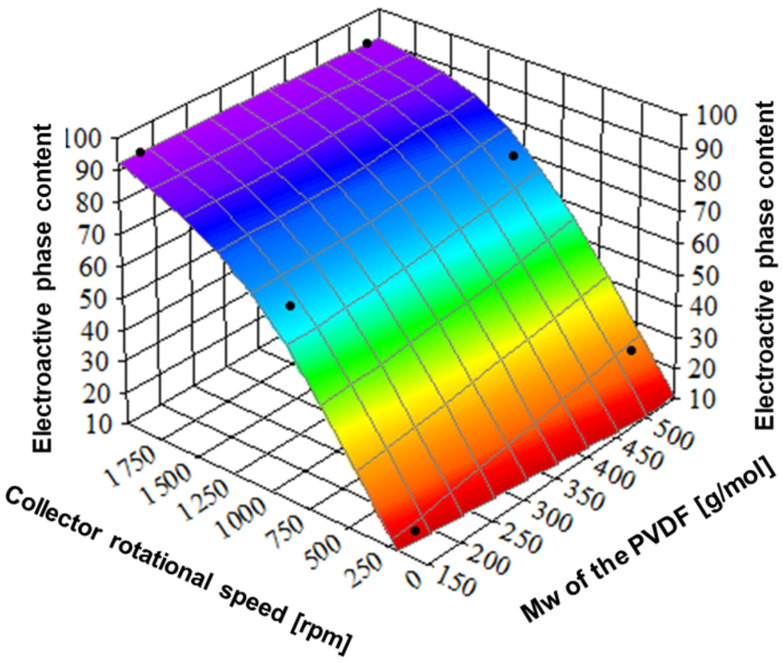
The relation between collector rotational speed, polymer molecular weight, and electroactive phase content. PVDF1—180,000 g/mol, collector speed—200 rpm; PVDF2—180,000 g/mol, collector speed—1000 rpm; PVDF3—180,000 g/mol, collector speed—2000 rpm; PVDF4—530,000 gm/mol, collector speed—200 rpm; PVDF5—530,000 g/mol, collector speed—1000 rpm; PVDF6—530,000 g/mol, collector speed—1000 rpm. All samples C_p_ = 20%, F_R_—1 mL/h (details in Table 1).

It is evident from Figure 11 that the effect of the collector’s rotational speed on the content of electroactive phases is strong. The electroactive phase fractions of samples from PVDF with lower molecular weight were determined to be 12, 65, and 92%, at 200, 1000, and 2000 rpm, respectively, and with higher molecular weight, 24, 72, and 91%, respectively. Such a strong increase in the content of electroactive phases with increasing collector rotational speed is related to an increase in stretching forces leading to better molecular alignment and orientation in the nanofibers. It is well known from the literature that molecular orientation induced by external fields like mechanical [44] promotes the formation of piezoelectric phases. The effect of molecular weight on the content of electroactive phases is evident only at the lowest rotational speed. The analysis of β and γ phase content (Figure 11, Table 1) indicates the gradual increase in γ phase content with collector rotational speed and kind of saturation of β phase content above 1000 rpm.

#### 2.2.2. Applied Voltage

**Figure 12:**
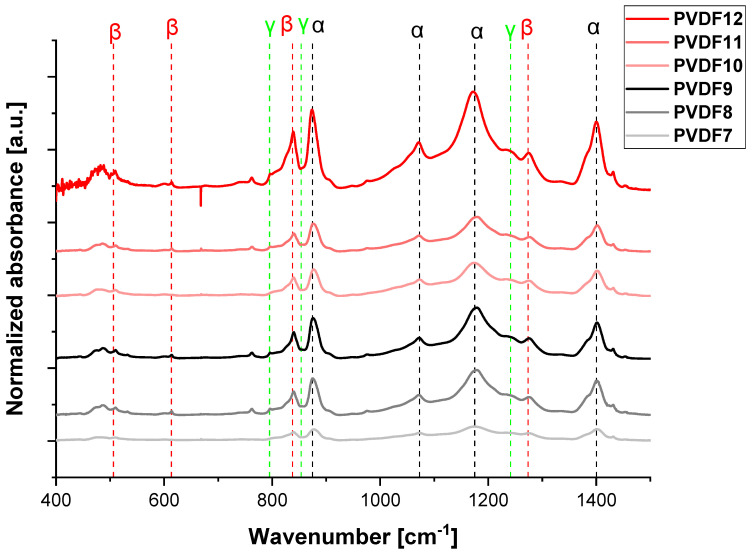
FTIR-ATR spectrum (400–1500 cm^−1^) of selected samples formed with various applied voltages. PVDF7—180,000 g/mol, applied voltage—15 kV; PVDF8—180,000 g/mol, applied voltage—20 kV; PVDF9—180,000 g/mol, applied voltage—25 kV; PVDF10—530,000 gm/mol, applied voltage—15 kV; PVDF11—530,000 g/mol, applied voltage—20 kV rpm; PVDF12—530,000 g/mol, applied voltage—25 kV. All samples: Cp = 20%, F_R_—1 mL/h, collector rotational speed—2000 rpm (details in Table 4).

**Figure 13:**
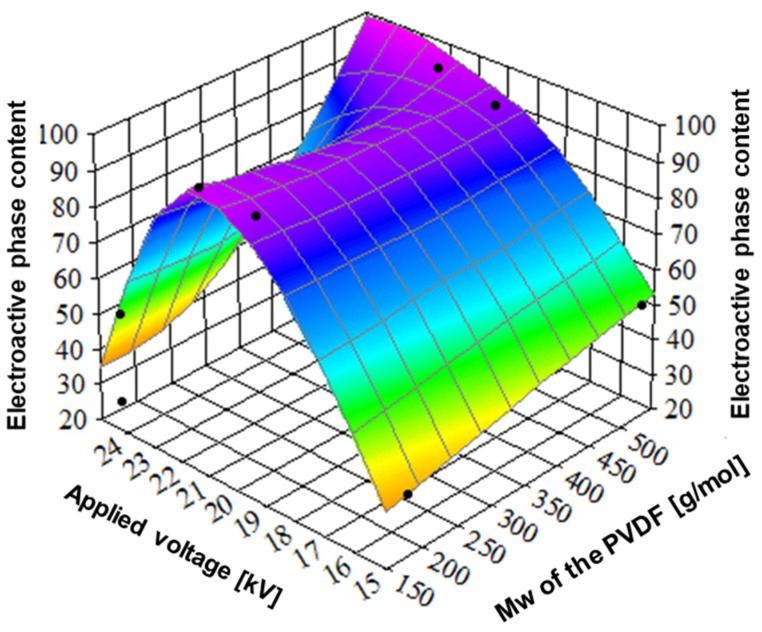
The relation between applied voltage, polymer molecular weight, and the sum of electroactive phase content. PVDF7—180,000 g/mol, applied voltage—15 kV; PVDF8—180,000 g/mol, applied voltage—20 kV; PVDF9—180,000 g/mol, applied voltage—25 kV; PVDF10—530,000 gm/mol, applied voltage—15 kV; PVDF11—530,000 g/mol, applied voltage—20 kV rpm; PVDF12—530,000 g/mol, applied voltage—25 kV. All samples: Cp = 20%, F_R_—1 mL/h, collector rotational speed—2000 rpm (details in Table 4).

In this section, the effect of applied voltage on the content of electroactive phases was investigated using the samples formed at the collector’s rotational speed allowing to obtain the highest content of electroactive phases. Figure 12 shows FTIR spectra of samples electrospun at 15 kV, 22 kV, and 25 kV. It is seen from Figure 13 that there is a maximum of the electroactive phases amount around 22 kV. Additionally, there is a weak effect of molecular weight on electroactive phase content; for instance, in the case of applied voltage 15 kV, the amount of electroactive phases is 37% and 52%, for low and high molecular weight, respectively.

During the electrospinning process of the PVDF, applied voltage causes the stretching and orientation of the polymer chains, promoting the formation of electroactive phases. The applied voltage is a factor of great importance in the effective stretching of the molecules and the formation of the electroactive phases [45]. It is evident from Figure 13 and Table 2 that the maximum of the phase content at 22 kV is reached by each of the electroactive phases, β, γ. The reduction in electroactive phase content above 22 kV is most probably related to disturbances of the electrospinning process and molecular orientation at the highest voltage [46]. Considering the positive effect of higher molecular weight on the formation of the electroactive phase, it can be concluded that a combination of higher applied voltage and higher molecular weight PVDF results in nanofibers with higher electroactive phase content while not exceeding the voltage at which the fibers are not effectively formed [47].

#### 2.2.3. The Flow Rate of the Solution

**Figure 14:**
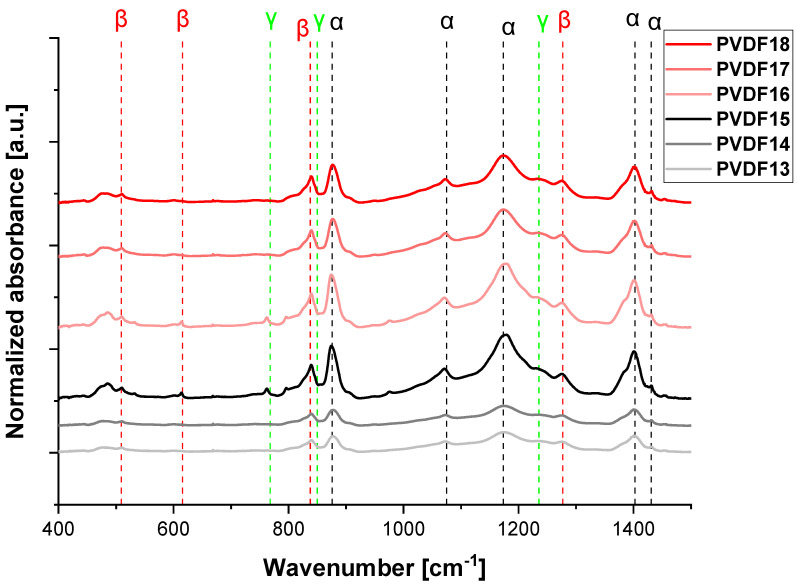
FTIR-ATR spectrum (400–1500 cm^−1^) of selected samples formed with various flow rates. PVDF13—180,000 g/mol, F_R_—0.5 mL/h; PVDF14—180,000 g/mol, F_R_—1.0 mL/h; PVDF15—180,000 g/mol, F_R_—1.5 mL/h; PVDF16—530,000 gm/mol, F_R_—0.5 mL/h; PVDF17—530,000 g/mol, F_R_—1.0 mL/h; PVDF18—530,000 g/mol, F_R_—1.5 mL/h. All samples: Cp = 20%, collector rotational speed—2000 rpm, applied voltage—20 kV (details in Table 4).

**Figure 15:**
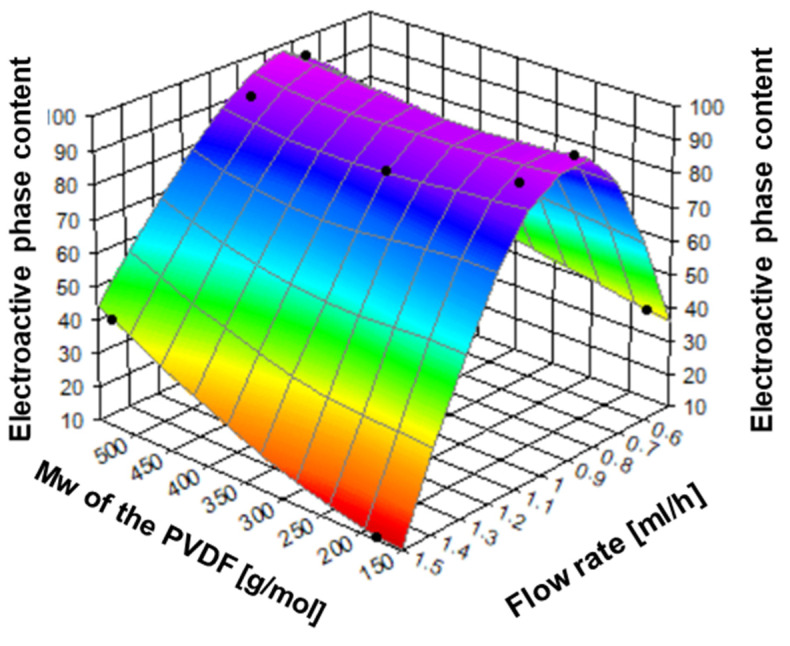
The relation between applied voltage, polymer molecular weight, and electroactive phase content. PVDF13—180,000 g/mol, _FR_—0.5 mL/h; PVDF14—180,000 g/mol, F_R—_1.0 mL/h; PVDF15—180,000 g/mol, F_R_—1.5 mL/h; PVDF16—530,000 gm/mol, F_R_—0.5 mL/h; PVDF17—530,000 g/mol, F_R_—1.0 mL/h; PVDF18—530,000 g/mol, F_R_—1.5 mL/h. All samples: Cp = 20%, collector rotational speed—2000 rpm, applied voltage—20 kV (details in Table 4).

Figure 14 shows the FTIR-ATR spectra for electrospun fibers using various flow rates—0.5 mL/h, 0.8 mL/h, and 1.5 mL/h. The lowest flow rate (0.5 mL/h) was insufficient to form a stable polymer jet due to excessive stretching (similar to high applied voltage) [48]. To summarize, when the stretching effect is too low, it is difficult to form the electroactive phases (F(β) + F(γ) in PVDF13 and PVDF16, 34% and 42%, respectively). When the flow rate increases, the polymer jet is more stable, and in effect, the amount of electroactive phases increases (F_R_ = 0.8 mL/h, F(β) + F(γ) = 89% and 96% in PVDF14 and PVDF17, respectively). It is caused by the stretching effect—when the flow rate increases, the stretching effect decreases [49]. Next, when the flow rate reaches the value of 1.5 mL/h or more, the increase in the flow rate lowers the stretching effect of the electric field, and the effect decreases the electroactive phase content (F(β) + F(γ) = 11% and 42% in PVDF15 and PVDF18, respectively) [50]. The changing of the flow rate affects the structure of the nanofibers and, more precisely, affects the stretching of the polymer solution. The phase content vs. flow rate has a maximum similar to the applied voltage and speed of the collector (Figure 15 and Table 3).

### 2.3. Piezoelectric Coefficient Measurements

**Figure 16:**
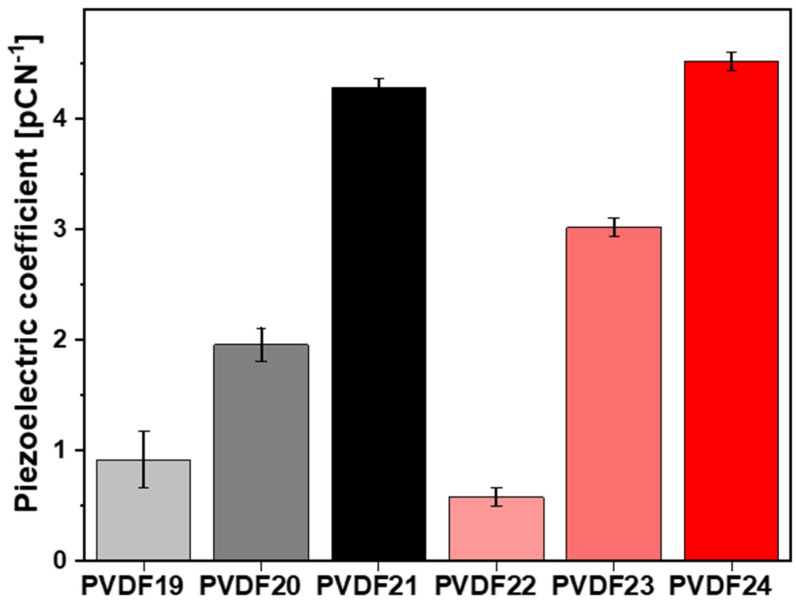
Results from d_33_ piezoelectric coefficient measurement of electrospun PVDF fibers. PVDF19—180,000 g/mol, collector rotational speed 200 rpm, PVDF20—180,000 g/mol, collector rotational speed—1000 rpm, PVDF21—180,000 g/mol, collector rotational speed—2000 rpm, PVDF22—530,000 g/mol, collector rotational speed—200 rpm, PVDF23—530,000 g/mol, collector rotational speed—1000 rpm, PVDF24—530,000 g/mol, collector rotational speed—2000 rpm. All samples: Cp—20%, applied voltage 22 kV, F_R_—0.8 mL/h (details in Table 4).

Measurements of the piezoelectric properties of the fibers with the highest electroactive phase content as a function of collector rotation speed and polymer molecular weight were performed (see Figure 16). Notably, regardless of the molecular weight used, the d_33_ response from the fibers increased with the rotations of the collector. It is seen that the effect of collector rotation speed is slightly larger for higher molecular weight samples, leading to higher d_33_ coefficients. For instance, the d_33_ piezoelectric coefficient for the highest rotational speed is 4.28 ± 0.08 for low molecular weight (PVDF21) and 4.52 ± 0.08 for high molecular weight (PVDF24). In contrast, the d_33_ piezoelectric coefficient for the lowest collector rotational speed is 0.9 ± 0.3 for low molecular weight (PVDF19), and 0.58 ± 0.08 for high molecular weight (PVDF22).

**Figure 17:**
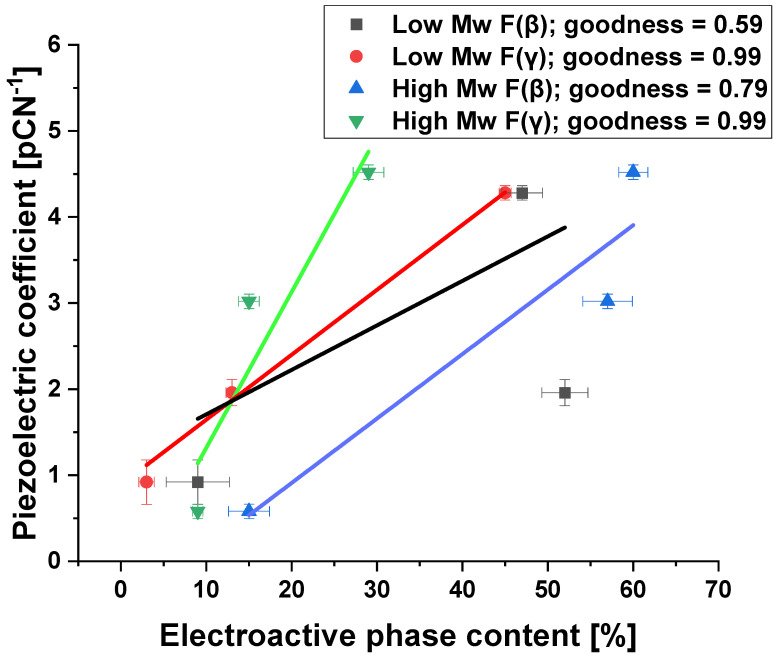
Dependence of the piezoelectricity coefficient d_33_ on the content of electroactive phases for low and high molecular weight PVDF.

An increase in the piezoelectric coefficient with collector rotational speed is caused by an increase in electroactive phase content. Figure 17 illustrates the dependence of the piezoelectric coefficient on β and γ phase content determined by FTIR. Despite the relatively large data scattering, it is evident, as expected, a strong dependence of piezoelectricity on the content of both electroactive phases [51].

It is worth noticing that the increase in the piezoelectric coefficient with the collector rotational speed results in an increase in the electroactive phase content. Moreover, the dependence of the piezoelectric coefficient on the content of electroactive phases β and γ was proved based on FTIR analysis. Despite some scatter in the data, a strong dependence of piezoelectricity on the content of both electroactive phases is visible. The values of the d_33_ coefficient vary depending on the specific material and its properties, such as crystal structure, degree of polymerization, and production conditions. There are studies in the literature in which the d_33_ coefficient plays a key role, but in, e.g., electronic applications and is [52,53]. In the designed scaffolds, the goal was to increase the d_33_ factor while maintaining the cytotoxicity of the material for tissue engineering applications, and there are few such detailed studies in the literature [54]. Moreover, human bone has piezoelectric properties lower than the designed materials and is at the level of 0.7–2.3 pC/N^−1^ [55,56,57]. Implanting a material with a higher piezoelectric coefficient d_33_ may result in faster regeneration of damaged bone.

### 2.4. In Vitro Study

**Figure 18:**
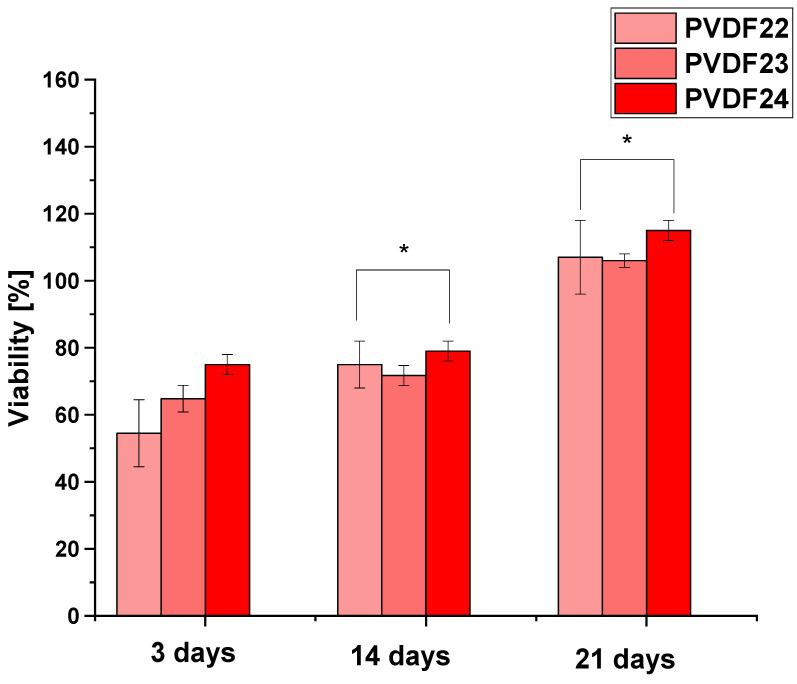
Cell viability of ADSC cultured on three types of PVDF samples different in rotational speeds of the collector applied—as described in Table 4, shown as the percentage of the values obtained in the control (cells cultured on tissue culture polystyrene). The data are presented as the mean value with standard deviation Statistical significance: * *p* < 0.05.

Cell viability was assessed for ADSC cultured on scaffolds with the highest number of electroactive phases formed from PVDF (Mw—530,000 g/mol). Three types of materials, obtained by using different rotational speeds of the collector, i.e., 200 rpm, 1000 rpm, and 2000 rpm, with the same applied voltage of 22 kV and flow rate of 0.8 mL/h were taken into observation. The results of the viability assay performed on those materials are presented in Figure 18. In all cases, the growing cell number in time was observed. The highest values were obtained for the scaffolds obtained by using 2000 rpm rotational speed. For this material viability of ≥70% of the control was found regardless of the time point in culture, which confirms its cytotolerance in accordance with the ISO 10993-5.

**Figure 19:**
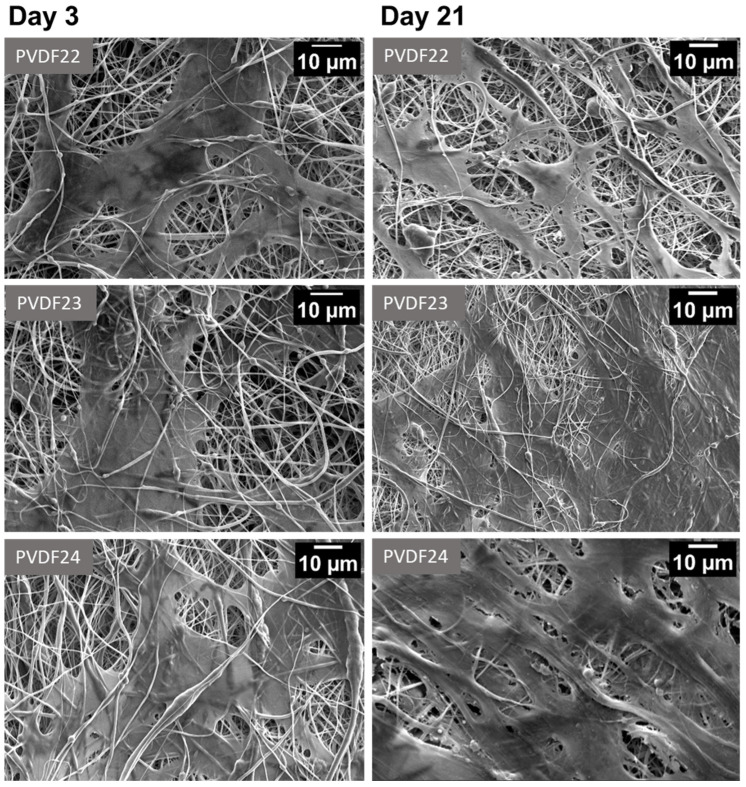
Representative SEM pictures of PVDF piezoelectric scaffolds cultured for 3, and 21 days. PVDF22—530,000 g/mol, collector rotational speed—200 rpm, PVDF23—530,000 g/mol, collector rotational speed—1000 rpm, PVDF24—530,000 g/mol, collector rotational speed—2000 rpm. All samples: Cp—20%, applied voltage 22 kV, F_R_—0.8 mL/h (details in Table 4).

ADSCs morphology on the PVDF22, PVDF23, and PVDF24 samples, as visualized by SEM on days 3 and 21, is presented in Figure 19. Cell attachment and spreading were observed in all cases, which confirms material cytocompatibility. The densest culture—consisting of cells and, most likely, also of the extracellular matrix produced by them—was observed on the PVDF24 surface on day 21.

Cell proliferation, attachment, and spreading may be difficult on the surface of the scaffolds obtained by electrospinning as compared to the surface of the culture dish, which served as a control, because of the availability of the points for the focal adhesion formation. Nevertheless, the obtained results confirm the cytocompatibility of the investigated scaffolds, especially the PVDF24, i.e., the scaffold obtained by using the rotational speed of the collector of 2000 rpm.

The development of nanostructured scaffolds with piezoelectric properties has shown promise in improving bone tissue regeneration [53]. Our research allows us to explore the issue of the formation and increase in the number of electroactive phases and their positive impact on the human adipose-derived stromal cells proliferation and also constitutes a good basis for determining the interactions of cells with the external environment (piezoelectric scaffold). In vitro, research on PVDF scaffolds is essential in the context of various fields of science [58], and PVDF is a material with unique properties that can be properly used to improve the lives of patients. Research on cell cultures on piezoelectric scaffolds concerns cell adhesion and proliferation [59], cytotoxicity, and the cultivation of piezoelectric scaffolds in the presence of ultrasound [60]. Recently, research has been conducted on the use of piezoelectric materials in the regeneration of vascularized bone [61]. However, there are also challenges associated with in vitro studies on PVDF scaffolds, such as controlling the electrospinning process to ensure a uniform and repeatable structure [62]. The quality of the scaffold formation is also important. Long-term studies are needed to determine the interactions of scaffolds with bone cells and the surrounding biological environment. Particularly noteworthy are ceramic nanoparticles with a high piezoelectricity coefficient (high d_33_), which, when dispersed in a polymer matrix, can have a positive effect on increasing the formation of piezoelectric phases while being biocompatible, which may constitute one of the systems with the most significant potential for BTE applications. In conclusion, in vitro studies on PVDF scaffolds have great potential in the field of regenerative medicine. However, further research is necessary to better understand the properties of these scaffolds and their potential clinical applications.

## 3. Materials and Methods

### 3.1. Materials

Poly(vinylidene fluoride) (PVDF) with various molecular masses, 180,000 g/mol and 530,000 g/mol (Merck, Rahway, NJ, USA) was used for the formation of the scaffold using electrospinning from *N*,*N*-Dimethylformamide (DMF), and acetone (Merck, USA) solutions.

All materials were analytical standard.

### 3.2. Electrospinning

The PVDF pellets from each polymer were dissolved in the solvent at a concentration of 20 wt % at 50 °C for 24 h until a visually homogeneous solution was formed. The electrospinning chamber (Fluidnatek LE-50, Bioinicia, Valencia, Spain) in a horizontal mode used for scaffold formation consisted of a syringe with the polymer solution, a needle 23G connected to the high-voltage supply with a positive potential, a pump, and a grounded collector. The fibers were collected as non-woven mats on a rotating drum (12 cm in length and 4 cm radius).

The process was provided at room temperature, 20% relative humidity, and 150 mm tip to the collector distance. The range of parameters was chosen to obtain a stable electrospinning process and continuous beadless fibers. The collected fiber mats were placed under the fume hood for 72 h for residual solvent evaporation. Table 4 describes the parameters used during electrospinning. Three sets of samples were analyzed from the perspective of the number of electroactive phases, morphology, and orientation. They were collected: (1) at various collector speeds, (2) at various applied voltages, and (3) at various flow rates. The effect of the applied voltage and of the flow rate was evaluated for the value of the collector speed 2000 rpm which led to the highest content of the electroactive phase. Then, 6 samples (PVDF19—PVDF24) were produced under favorable conditions for the formation of electroactive phases (applied voltage—22 kV, F_R_—0.8 mL/h) and tested to determine the piezoelectric coefficient (d_33_). Finally, 3 samples (PVDF22—PVDF24) with the highest number of electroactive phases (Mw—530,000 g/mol), formed at different collector rotational speeds (200 rpm, 1000 rpm, and 2000 rpm), were selected for in vitro tests.

### 3.3. Scanning Electron Microscopy (SEM)

Characterization of fiber morphology was performed using scanning electron microscopy (SEM, JSM-6010PLUS/LV InTouchScope™, JEOL, Tokyo, Japan) at an acceleration voltage of 10 kV. Prior to analysis, a layer of gold was applied to all samples. Quantitative microstructure analysis was performed using ImageJ software with the Fiji distribution plugin using Gaussian function approximation [63]. Fiber diameter was measured on 50 fibers for each fiber mat sample. The fiber diameter distribution was analyzed with the Gaussian fitting, also providing the goodness of fit using ImageJ software (1.52q software version).

The effect of the rotational speed of the collector on the fibers’ orientation in the scaffold was determined using the directionality plugin to the ImageJ software. The orientation patterns were determined using the Fourier Transform on sections of the SEM images. The orientation distribution was approximated using the Gaussian function with Origin 2021b software. The degree of fiber alignment was additionally estimated using the full-width at half maximum (FWHM) of the Gaussian used for approximation of the orientation distribution. FWHM was averaged on five images for each sample.

The orientation factor was calculated using the anisotropy index *α* (*α* = 1 for perfect alignment, and *α* = 0 for random distribution), (Equations (1) and (2)) [64].

(1) $$\Omega =\frac{1}{I_{tot}}\sum I_{i}\left|\begin{array}{cc} {cos}^{2}\theta_{i} & {sin\theta }_{i}{cos\theta }_{i}\\ sin\theta_{i}cos\theta_{i} & {sin}^{2}\theta_{i} \end{array}\right|\;$$

(2) $$\alpha =1-\lambda_{1}/\lambda_{2}$$

where *Ω* is the orientation matrix with eigenvalues of *λ*_1_ and *λ*_2_, *I_tot_*—is the sum of the nanofiber length (nm), *I_i_*—length of a single *i*-th nanofiber (nm), *θ_i_*—the angle between the nanofiber axis and the x-axis (the direction perpendicular to the collector axis). The lengths of the nanofibers and the angles were determined from the SEM images using the Directionality plugin to the ImageJ software.

Total average porosity (*p*) was calculated as in our earlier research (Equation (3)) [65]:

(3) $$p=\frac{V_{t}-V_{f}}{V_{t}}=\left(1-V_{f}\times \frac{\rho }{m_{t}}\right)=(1-\frac{m_{f}}{m_{t}})$$

In this equation, *m_t_* represents the theoretical mass of the solid sample, calculated as the product of the volume occupied by a patch *V_t_* and the density of PVDF (1.75 g/cm^3^) while *V_f_* and *m_f_* denote the actual sample volume and mass, respectively. The average pore size *(p*) was determined using the following formula (Equation (4)):

(4) $$P=\frac{2D}{\left(1-p\right)}$$

*D* represents the mean fiber diameter, and (1 − *p*) denotes the average total projected area of fibers per unit area, where *p* is approximately the total porosity.

### 3.4. Fourier Transform Infrared Spectroscopy (FTIR)

The content of the electroactive phases in the nonwoven scaffolds was determined using Fourier transform infrared spectroscopy (FTIR-ATR, Bruker Vertex 70, Billerica, MA, USA). The results presented are representative for five independent specimens and runs. The specimens were investigated from 400 cm^−1^ to 4000 cm^−1^ with a resolution of 2 cm^−1^, which summarizes 32 scans.

*F_EA_*, the relative content of two piezoelectric phases, β and γ, was determined in accordance to [42,66] using equation:

(5) $$F_{EA}=\frac{I_{840*}}{\left(\frac{K_{840*}}{K_{763}}\right)\times I_{763}+I_{840*}}\times 100\%$$

where *I*_763_ and *I*_840*_ are the intensities of the bands at 763 cm^−1^ and 837–841 cm^−1^, assigned to the α phase, and to both β and γ phases together, respectively. These absorption bands follow Beer–Lambert law with absorption coefficients of *K*_840*_ = 7.7 × 10^4^ cm^2^ mol^−1^, and *K*_763_ = 6.1 × 10^4^.

The absorbance of the peak area or peak height at bands at 1234 cm^−1^ and 1275 cm^−1^ was used to quantify individual β and γ phases content, *F*(*β*) and *F*(*γ*). The procedure used was the calculation of the peak-to-valley height ratio between the peaks around 1234 cm^−1^ and 1275 cm^−1^, and their nearest valleys.

(6) $$F\left(\beta \right)=F_{EA}\times \left(\frac{∆H_{\beta^{\prime }}}{∆H_{\beta^{\prime }}+∆H_{\gamma^{\prime }}}\right)\times 100\%$$

(7) $$F\left(\gamma \right)=F_{EA}\times \left(\frac{∆H_{\gamma^{\prime }}}{∆H_{\beta^{\prime }}+∆H_{\gamma^{\prime }}}\right)\times 100\%$$

where Δ*Hβ′* is the absorbance difference (height difference) between the peak, 1275 cm^−1^, and the nearest valley, around 1260 cm^−1^, and Δ*Hγ′* is the peak, around 1234 cm^−1^ and the nearest valley, around 1225 cm^−1^.

### 3.5. Piezoelectric Coefficient Measurements

The piezoelectric coefficients of the electrospun PVDF scaffolds were examined using a d_33_ meter (YE2730A d_33_ meter, Sinocera, Shanghai, China). This instrument is specifically designed for directly measuring the d_33_ values of piezoelectric materials. For testing, an 8 nm 80Au−20Pd layer (Q150RS, Quorum Technologies, Laughton, East Sussex, UK) was applied as an electrode on both sides of the PVDF fibers. Five measurements were conducted for each sample, and the average d_33_ value was calculated. To account for potentially low piezoelectric values of fibers resulting from the high porosity of the prepared materials, a reference sample was also tested to validate the instrument. The reference sample was a piezoelectrically poled PVDF film 110 μm thick, with a piezoelectric coefficient stated by the supplier ranging from 23 to 28 pCN^−1^, obtained from PolyK (Philipsburg, PA 16866, USA). The sample was used as received after a 4 × 4 cm square was cut out from the polymer film. Prior to the measurements on fibers, the instrument was validated using the reference sample, which yielded a value of 25.6 ± 2.3 pCN^−1^, falling within the manufacturer’s specified range of 23–28 pCN^−1^.

### 3.6. In Vitro Tests

Human adipose-derived stromal cells (ADSCs) were obtained according to established protocol as previously described [67] and cultured on the piezoelectric PVDF scaffolds electrospun with different collector rotational speeds (200, 1000, and 2000 rpm). First, PVDF samples were cut and sterilized by radiation. Next, for the cell viability test, piezoelectric scaffolds were placed in a 96-well culture plate (six samples for each type of material) and ADSCs were seeded directly on the surface of the scaffolds in a density of 4 × 10^3^ cells per well. Cells seeded at the bottom of culture dish (6 wells) served as a control. For SEM observation, scaffolds were inserted into a 24-well culture plate (two samples per group) and seeded with a total of 5 × 10^4^ cells per well. Cells were cultured in an osteogenic differentiation medium, composed of DMEM with 10% fetal bovine serum (FBS) and 1% antibiotic–antimycotic, supplemented with 10 nM dexamethasone, 3 mM NaH_2_PO_4_, and 50 μg/mL ascorbic acid 2-phosphate [68]. All specimens were incubated at 37 °C in 5% CO_2_ for 21 days.

PrestoBlue assay (Thermo Fisher Scientific, Basingstoke, UK) for studying cellular metabolism activity was performed after 3, 14, and 21 days. First, the culture medium was removed, and cells were rinsed with PBS solution. Next, PrestoBlue working solution was applied and samples were placed in the incubator for 2 h. After incubation, solutions were transferred to new 96-well plate. Fluoroskan Ascent was used to measure the emission of the light at wavelengths 620 nm and 530 nm. Results were evaluated in comparison to the metabolic activity of the cells seeded on TCP (Tissue Culture Plastic, control 100%).

The observations of fibers and cell morphology were conducted using scanning electron microscopy (SEM, JSM-6010PLUS/LV InTouchScope™, JEOL, Tokyo, Japan) after 3 and 21 days. First, samples were properly prepared, washed with PBS, and next, were fixed with 2.5% glutaraldehyde in PBS overnight. The process of dehydration was conducted using water and ethanol (30, 40, 50, 60, 70, 80, 90, 100 (×2)% *v*/*v*). Then, ethanol/HDMS (Sigma Aldrich, Burlington, MA, USA) solutions were used (2:1 and 1:2 *v*/*v*). The last stage was to rinse samples with pure HDMS and leave them overnight.

### 3.7. Statistical Analysis

The data were additionally utilized to assess their statistical significance. The statistical analysis of viability data was conducted for *p* < 0.05 using GraphPad Prism 8.0.1 Software (GraphPad, Boston, MA, USA). A two-way ANOVA with Tukey’s multiple comparisons test was conducted as necessary. A *p*-value below 0.05 was deemed statistically significant. Notations of “*” were assigned as 0.01 < *p* < 0.05, “**” was assigned as 0.001 < *p* < 0.01, and “***” was assigned as *p* < 0.001.

## 4. Conclusions

This study aimed to better understand to fundamental process of forming the polar phases in PVDF. The most important result is a demonstration of the effect of selected electrospinning parameters—rotational collector speed, the solution flow rate, and the applied voltage on polymorphs content of two PVDF types differing in molecular weight (180,000 g/mol and 530,000 g/mol). It is evident that in the case of rotational collector speed, there is a continuous increase in electroactive β and γ phase content, in addition to the rise of fiber orientation and reduction in fiber diameter. In the case of the applied voltage and solution flow rate, there is a maximum of electroactive phase content at a particular parameter’s value. Therefore, the electroactive phase content is possible by optimizing the processing parameters. The maximum electroactive phase content equal to 92% was obtained at a rotational collector speed of 2000 rpm, voltage of 22 kV, and a flow rate of 0.8 mL/h. An increase in electroactive phase content is responsible for the observed increase in piezoelectric d_33_ coefficient, which could be crucial for cells considering the further perspective of using this kind of scaffold in regenerative medicine. Cell culture using human-adipose-derived stromal cells confirmed the non-cytotoxic nature of scaffolds. Additionally, it was observed that the cell viability increases not only with cultivation time but also for fibers collected with the highest rotational speed, with the best fiber orientation and the largest content of electroactive phases.

## Figures and Tables

**Table 1:** Electroactive phase content in PVDF scaffolds.

Sample ID	F(α) (%)	F(β) + F(γ) (%)	F(β) (%)	F(γ) (%)
PVDF1	88 ± 0.2	12 ± 1.2	9 ± 3	3 ± 4
PVDF2	35 ± 1.4	65 ± 0.7	52 ± 6.1	13 ± 3.3
PVDF3	8 ± 2.2	92 ± 4	47 ± 2	45 ± 4.8
PVDF4	76 ± 3	24 ± 0.4	15 ± 0.6	9 ± 4.2
PVDF5	28 ± 1.9	72 ± 2.9	57 ± 0.9	15 ± 1.3
PVDF6	11 ± 3.3	91 ± 6.3	60 ± 2	29 ± 1.2

**Table 2:** Electroactive phase content in PVDF scaffolds.

Sample ID	F(α) (%)	F(β) + F(γ) (%)	F(β) (%)	F(γ) (%)
Applied Voltage [kV]	15 kV			
PVDF7	63 ± 2	37 ± 0.4	23 ± 2.7	14 ± 2.9
PVDF10	48 ± 0.6	52 ± 2.9	28 ± 0.6	24 ± 0.9
Applied Voltage [kV]	22 kV			
PVDF8	8 ± 3	93 ± 1.4	48 ± 0.7	45 ± 1.1
PVDF11	11 ± 6.1	95 ± 6.3	45 ± 4	50 ± 0.9
Applied Voltage [kV]	25 kV			
PVDF9	78 ± 2	22 ± 0.6	15 ± 0.9	6 ± 3.4
PVDF12	63 ± 0.9	47 ± 0.7	26 ± 1.4	21 ± 1.8

**Table 3:** Electroactive phase content in PVDF scaffolds.

Sample ID	F(α) (%)	F(β) + F(γ) (%)	F(β) (%)	F(γ) (%)
Flow Rate	0.5 mL/h			
PVDF13	63 ± 2	37 ± 0.4	23 ± 2.7	14 ± 2.9
PVDF16	48 ± 0.6	52 ± 2.9	28 ± 0.6	24 ± 0.9
Flow Rate	0.8 mL/h			
PVDF14	7 ± 3	93 ± 1.4	48 ± 0.7	45 ± 1.1
PVDF17	3 ± 6.1	97 ± 6.3	47 ± 4	50 ± 0.9
Flow Rate	1.5 mL/h			
PVDF15	89 ± 2	11 ± 0.6	9 ± 0.9	7 ± 3.4
PVDF18	58 ± 0.9	42 ± 0.7	27 ±1.4	15 ± 1.8

**Table 4:** Sample identification.

Sample ID	Molecular Weight [g/mol]	Cp [%]	Applied Voltage [kV]	Flow Rate [ml/h]	Collector Speed [rpm]
PVDF1	180,000	20	20	1	200
PVDF2					1000
PVDF3					2000
PVDF4	530,000				200
PVDF5					1000
PVDF6					2000
PVDF7	180,000		15	1	2000
PVDF8			20		
PVDF9			25		
PVDF10	530,000		15		
PVDF11			20		
PVDF12			25		
PVDF13	180,000		20	0.5	2000
PVDF14				1	
PVDF15				1.5	
PVDF16	530,000			0.5	
PVDF17				1	
PVDF18				1.5	
PVDF19	180,000		22	0.8	200
PVDF20					1000
PVDF21					2000
PVDF22	530,000				200
PVDF23					1000
PVDF24					2000

## Data Availability

Data are contained within the article.
